# Supplementary material for: Integrated multi-port circulators for unidirectional optical information transport
Source: Sci Rep. 2017 May 18;7:2129. doi: 10.1038/s41598-017-02340-9 (PMC5437014; doi:10.1038/s41598-017-02340-9)
Supplement: Supplementary file 1 — Supplementary Information [file 41598_2017_2340_MOESM1_ESM.doc]

**Integrated multi-port circulators for unidirectional optical information transport**

Parinaz Aleahmad, Mercedeh Khajavikhan, Demetrios Christodoulides, and Patrick LiKamWa

CREOL, The College of Optics and Photonics, University of Central Florida, Orlando, Florida 32816, USA

1. **Theory and beam propagation analysis of a nonlinear unidirectional coupler**

We here analyze wave transport in a lossy nonlinear directional coupler comprised of two identical waveguide elements. To understand the light dynamics in this system (that plays a central role in our circulator design), we use the following model as obtained from coupled-mode theory:


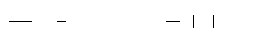


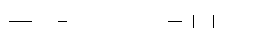


(1.1)

In the above equations,
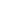
 represent modal field amplitudes,
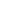
 stands for the intensity loss coefficient,
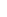
 is the coupling constant and
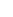
 denotes the nonlinear Kerr coefficient of the material. More information concerning this configuration can be found in references [1-4].

In addition to coupled mode equations, in this study we have utilized numerical beam propagation methods (BPM) to provide a more accurate description of the nonlinear directional coupler.

In our simulations, the defocusing nonlinear coefficient is taken to be
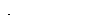
 (as obtained experimentally) while the loss constant is
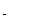
, again determined experimentally. All simulations were performed at 1550 nm. Figure S1. shows the intensity distribution in this system when excited with low intensity (left) and high intensity signals. Under low intensity conditions, the signal is routed to the adjacent waveguide. Conversely in the high intensity regime, the signal remains in the originally excited channel since it is capable of detuning the system with the help of the defocusing nonlinearity. The differential in intensity transmission (within the original port) under high and low intensity conditions, is in our case in excess of 20 dB. This is responsible for the unidirectional transport as described in the main text.

1. **Fabrication process**

The sample used in this study was ultrasonically cleaned with acetone to remove debris and organic residues from the top layer, followed by rinsing with isopropyl alcohol (IPA) and deionized (DI) water. The sample was then immersed in buffered oxide etch (BOE) to remove any native oxide from the surface. Subsequently, a 30 nm thick layer of SiN was deposited using plasma-enhanced chemical vapor deposition (PECVD) which served as a capping layer that inhibited the intermixing process. The SiN was grown at a temperature of 250^o^C with an RF power of 100 W, at 900 mTorr residual pressure with a gas flow rate of 180 sccm of 2% silane pre-diluted with nitrogen, 6 sccm of NH_3_, 200 sccm of N_2_ and an additional 219 sccm of He to control the stress behavior of the film, making it resistant to cracking during the rapid thermal annealing (RTA) treatment. The regions of the multiple quantum well layer that constitute the nonlinear decoupler (ND) are delineated using positive photoresist S1805 and contact mask lithography followed by reactive ion etching of the other areas of the SiN film. To obtain maximum optical gain in the SOA segments, the bandgap energy of the MQWs had to be slightly red shifted. This was achieved using a capping layer of Si-rich SiN film. That film was grown by PECVD using the same recipe as that for the inhibitor SiN, with the only difference being that the flow rate of NH_3_ was reduced from 6 sccm to 4.5 sccm. Photolithography and reactive ion etching was again used to delineate the SOA and ND sections. Finally a film of SiO_2_ 200 nm thick, was deposited over the entire sample to serve as a promoter for MQW intermixing in the regions that were not covered by SiN. The SiO_2_ was grown under similar conditions as for the SiN film, with 400 sccm of SiH_4_ and 200 sccm of N_2_O and the notable absence of NH_3_. The sample was then treated by RTA at a temperature of 775^o^C for 30 s. The pattern of waveguiding structures was then transferred through the SiN/SiO_2_ capping layers by photolithography and the RIE process using O_2_ and CF_4_ gasses. The remaining photoresist layer was then stripped using acetone and the upper InP layer was etched using room temperature ICP-RIE at a residual pressure of 5 mTorr with the ICP power at 200 W and the RF plasma power kept at 100 W. The gases included CH_4_ at 17 sccm, H_2_ at 8 sccm and Ar at 5 sccm. Another layer of SiN was then deposited on the entire sample and windows were opened to allow deep etching of the regions containing the 90° waveguide bends all the way through the MQW layer. When the ICP-RIE process was complete, all the covering dielectric layers were removed using RIE in a plasma of CF_4_ and O_2_. The top surface was planarized by spin-coating the sample with benzocyclobutene (BCB) resin (commercially known as Cyclotene), and baking it in a nitrogen oven at 250^o^C for one hour and then etching it back until just about 200 nm of the top InGaAs layer was uncovered. P-type contact metals consisting of Ti, Zn and Au were then deposited on top of the waveguides that operate as SOA sections. The samples were annealed in a nitrogen atmosphere at 430ºC for 30 s to diffuse the metal contacts into the surface of the semiconductor. The sample was then lapped and polished to a final thickness of 120
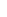
m before the n-type contact metals (Ni, Ge, Au) were deposited on the substrate side of the devices followed by a second annealing. The device was finally cleaved to an overall length of 6.5 mm.

1. **Characterization measurements**

## 3.1 Optical loss measurements

The bandgap energy of the MQW in the passive regions (excluding the ND and SOA sections), is tuned to a shorter wavelength with respect to the signal wavelength so as to minimize material absorption losses. In order to verify that the MQW intermixing process was indeed successful, we have measured the optical losses of ridge waveguides fabricated in both bandgap shifted and non-shifted regions. By varying the wavelength of the probing laser, the transmitter power in these waveguides was recorded in order to observe the Fabry-Perot fringes resulting from reflections at the two cleaved facets. The waveguide attenuation coefficient is then obtained from the visibility factor *V,* of the fringes through:


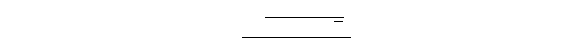


where the reflectivity of each facet is given by:
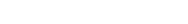
 and the fringe visibility
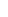
 is given by:


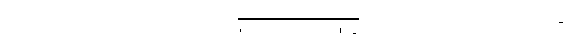


This scheme is suitable for waveguides with an attenuation constant that is less than 10 cm^-1^. However, as the wavelength of the laser is varied toward the shorter end of the spectrum, the waveguide attenuation increases and the fringe visibility becomes too small to be reliably measured. Based on fringe visibility measurements, we were able to extract both the attenuation coefficient α, and the input power
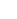
 launched into the waveguide. By simply measuring, the output power
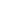
 (for wavelengths where the fringe visibility is small), we can deduce the attenuation coefficient directly from:


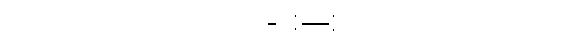


Using this technique, we have measured the wavelength dependence of the loss coefficient in ridge waveguides (averaged over 10 different devices) fabricated in the blue and red shifted regions respectively.

Finally, we have characterized the optical losses introduced by the total internal reflection mirrors designed to provide 90° waveguide bends at the center of the re-routing structure. For this measurement, we have fabricated a waveguide system that contains two 90° turning mirrors, alongside a perfectly straight waveguide as shown in figure S2 (a) and (b). Assuming that the input powers and the waveguide attenuation coefficients are identical, we were able to extract the reflectivity
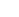
 for each waveguide mirror from:


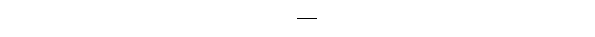


where
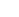
 and
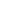
 are the measured optical powers at the outputs of the straight waveguide arrangement and that containing two mirrors respectively. From these measurements we deduced that the reflectivity of each mirror is approximately 95%.

## 3.2 Characterization of the nonlinear response associated with the InGaAsP quantum wells

The effective nonlinear Kerr coefficient, is measured by monitoring the phase change acquired by light when passing through this system as a function of the optical power density. To do so, we have assembled a free space Mach-Zehnder interferometer as shown in figure S3. The nonlinear phase change was measured according to:


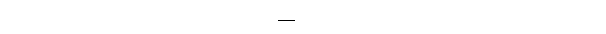


where
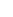
 is the effective nonlinear Kerr coefficient,
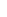
 is the wavelength, and
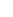
 is the effective length of the sample (that accounts for losses) . Here,
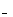
 represents a change in the optical intensity,
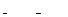
, where
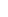
 is the effective cross-sectional area of the ridge waveguide mode. Therefore, the nonlinear refractive index is obtained from


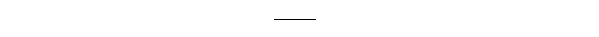


where
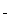
 is the observed number of fringes moved in response to a change of the optical power
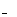
.

**3.3 Gain calibration and semiconductor optical amplifier device characterization**

To characterize the optical gain in the MQW system, we have fabricated different semiconductor optical amplifiers (SOAs) with cleaved facets. Following the same method described in section 3. 1, an SOA device (below the lasing threshold) functions as a Fabry-Perot cavity and consequently the gain coefficient can be obtained directly from the fringe visibility [5, 6] using


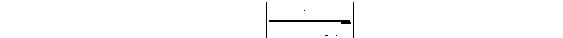


where
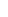
 is the visibility of the fringes and
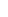
 is the reflectivity of each waveguide facet. When
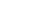
, this signifies the critical visibility for which the net gain is zero. The P-I curve of this laser device was used to obtain the threshold current, measured to be around
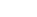
. In order to obtain the optical gain of the SOA device for currents higher than
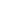
, the feedback from the cleaved facets was removed by depositing a 200 nm thick layer of SiN on each end. The absolute value of the gain coefficient was then evaluated using the calibrations from the net gain values obtained below and slightly above the transparency point.

**Figures:**


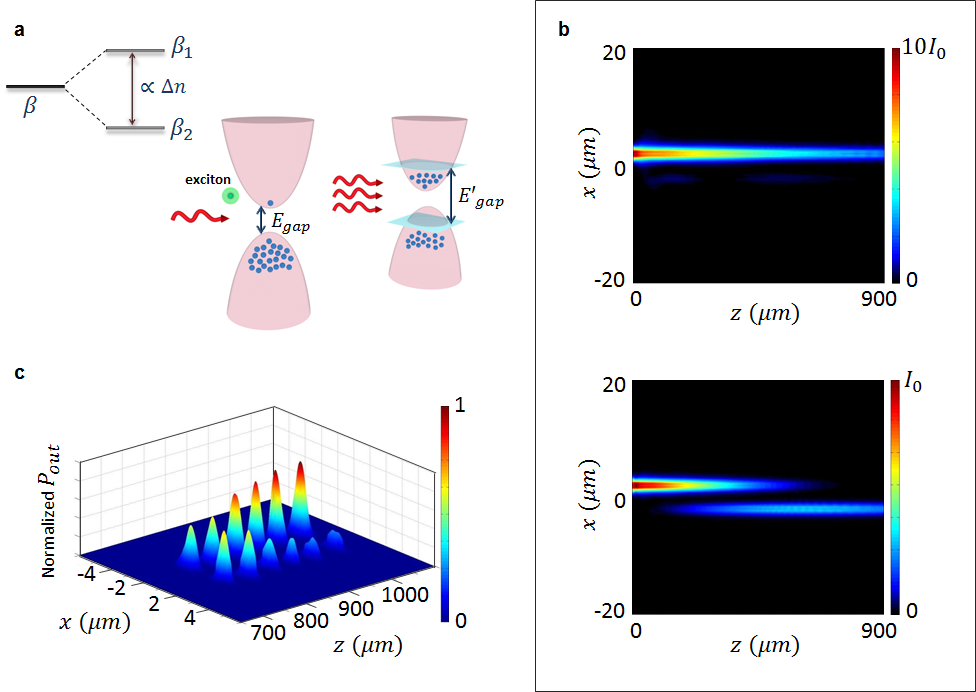


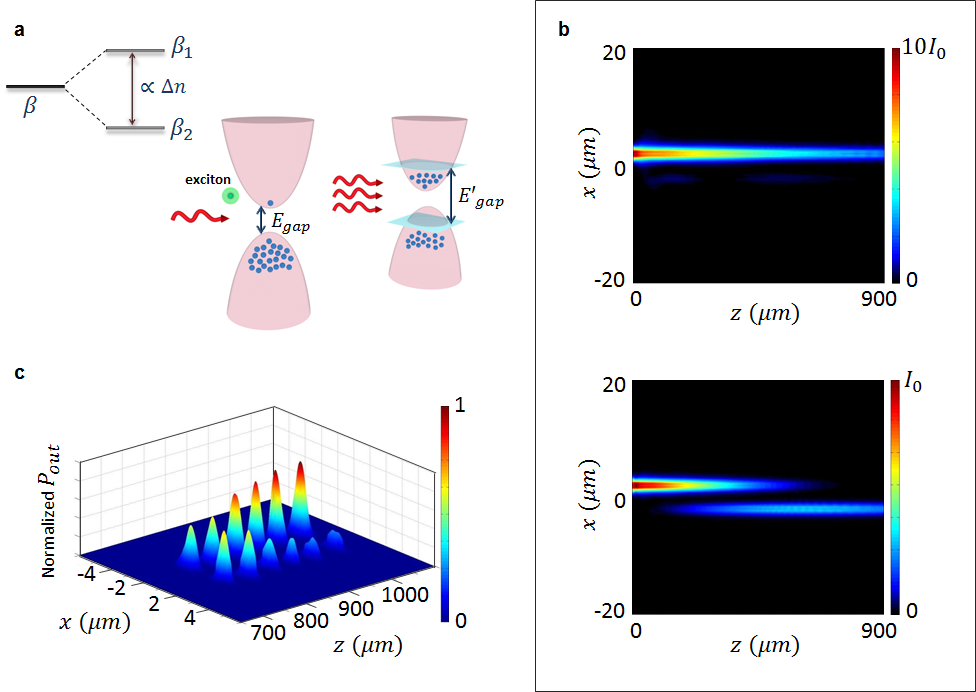


**Figure S1.** BPM simulations of a lossy nonlinear directional coupler using the parameters extracted experimentally


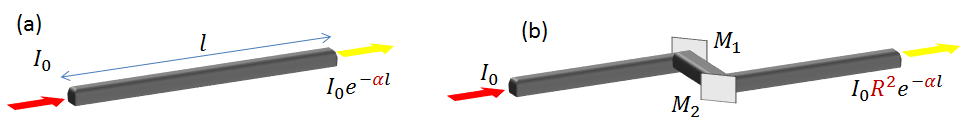


**Figure S2. a.** Intensity attenuation of an optical pulse propagating along a single waveguide versus, **b.** a waveguide involving two (total internal) reflection mirrors.


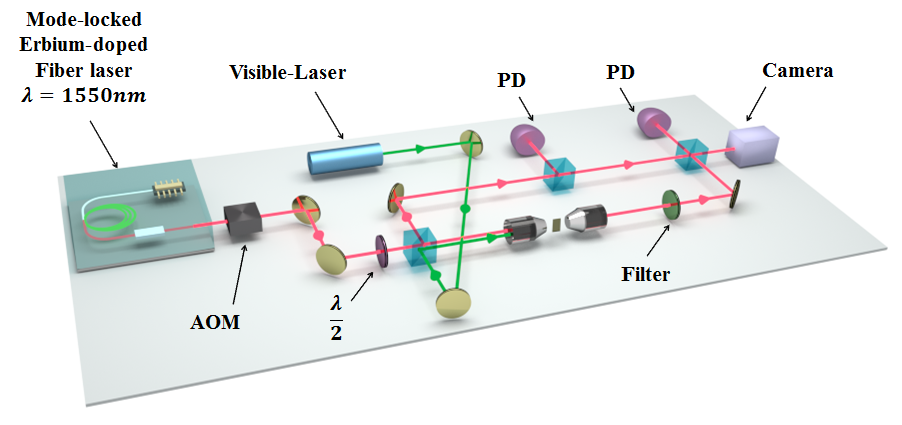


**Figure S3.** Free-space Mach Zehnder interferometer used for nonlinear measurements.

**References:**

1. Daino, B., G. Gregori, and S. Wabnitz, *Stability analysis of nonlinear coherent coupling.* Journal of Applied Physics, 1985. **58**(12): p. 4512.

2. LiKamWa, P., et al., *All optical multiple-quantum-well waveguide switch.* Electronics Letters, 1985. **21**(1): p. 26.

3. Das, U., Y. Chen, and P. Bhattacharya, *Nonlinear effects in coplanar GaAs/InGaAs strained-layer superlattice directional couplers.* Applied Physics Letters, 1987. **51**(21): p. 1679.

4. Friberg, S.R., et al., *Ultrafast all-optical switching in a dual-core fiber nonlinear coupler.* Applied Physics Letters, 1987. **51**(15): p. 1135.

5. Hakki, B.W. & Paoli, T.L. Gain spectra in GaAs double−heterostructure injection lasers. *J. Appl. Phys.* **46**, 1299 (1975).

6. Choy, W.C.H. & Chan, K.S. Theoretical analysis of diffused quantum-well lasers and optical amplifiers. *IEEE J. Sel. Topics Quantum Electron.* **9**, 698-707 (2003).
